# Huddle Up! A Comprehensive Approach to Improving Interdisciplinary Huddle in a VA Geriatric Primary Care Clinic

**DOI:** 10.1093/geroni/igaa057.042

**Published:** 2020-12-16

**Authors:** Juliana Wilking-Johnson, Joleen Sussman

**Affiliations:** 1 University of Colorado School of Medicine, Aurora, Colorado, United States; 2 Rocky Mountain Regional VAMC, Aurora, Colorado, United States

## Abstract

Spending time in brief meetings or “huddles” is associated with greater job satisfaction and less burnout, especially when team members have mutually agreed upon goals and can participate in decision-making. However, huddles are often unproductive and may have opposite the intended effect if not tailored to the specific team involved. We sent a survey to all members of our VA Geriatric primary care team (including geriatric medicine fellows, social work, psychology, audiology, pharmacy, faculty and support staff) asking them to rate our huddle’s impact on their stress level, efficiency, learning, preparedness and feeling supported at work. Responders indicated if they received needed information and if they understood what information was needed from them. We then held a team meeting to establish mutually agreed upon goals, expectations and organization of huddle, which were then reinforced with visual and timer reminders. After 6 weeks utilizing the new format, we administered a post intervention survey assessing the impact of the change. The initial survey revealed that the geriatric medicine fellows had worse ratings than other trainees, staff, and faculty. Fellows were more likely to say that they did not know what information was needed from them; and they did not receive information needed from others. The follow up survey showed improvement in all scores among geriatrics medicine fellows and allied health professionals, including 100% of respondents indicating they receive needed information. Overall, comments regarding the intervention were positive, demonstrating that a structured, organized huddle tailored to a specific team, can be beneficial.

